# Functional beverages improve insulin resistance and hepatic steatosis modulating lysophospholipids in diet‐induced obese rats

**DOI:** 10.1002/fsn3.2162

**Published:** 2021-02-06

**Authors:** Julio C. Rubio‐Rodríguez, Rosalia Reynoso‐Camacho, Nuria Rocha‐Guzmán, Luis M. Salgado

**Affiliations:** ^1^ CICATA‐Querétaro Instituto Politécnico Nacional Querétaro México; ^2^ Research and Graduate Studies in Food Science Faculty of Chemistry Autonomous University of Queretaro Queretaro Mexico; ^3^ Departamento de Ingenierías Química y Bioquímica Research Group on Functional Foods and Nutraceuticals TecNM/Instituto Tecnológico de Durango Durango México

**Keywords:** functional beverage, insulin resistance, lipids metabolism, lysophospholipids, NAFLD (nonalcoholic fatty liver disease)

## Abstract

Hypercaloric beverages increase the prevalence of insulin resistance and nonalcoholic fatty liver disease (NAFLD), diets with polyphenolic compounds improved these alterations. The study aimed to evaluate the effect of the consumption of three functional beverages (prepared with: Roselle, green tea, cinnamon, Malabar tamarind, and peppermint in different proportions) on insulin resistance and NAFLD and their relation to liver phospholipid regulation in Wistar rats fed with a high‐fat and fructose (HFF) diet. The consumption of beverages showed lower liver triglycerides compared to HFF control group, being the called beverage B the successful triggering up to 30.1%. The consumption of functional beverages improved insulin resistance and decreased the abundance of LysoPC (20:2), LysoPC (16:0), LysoPC (14:0), LysoPE (18:0), LysoPC (15:0), and LysoPC (20:1), with beverage C being the one with the meaningful effect. The results indicate that the functional beverage consumption improves insulin resistance, and decrease the degree of NAFLD, these through modifications of lysophosphatidylcholines, and lipids metabolism.

## Graphical abstract

Hypercaloric beverages increase the prevalence of insulin resistance and nonalcoholic liver disease. The study evaluated three functional beverages, prepared with: Roselle, green tea, cinnamon, Malabar tamarind, and peppermint, on insulin resistance and NALFD. The results indicate that the consume of the formulated beverages improve the insulin resistance, and decrease the degree of NALFD.

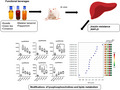

## 1 INTRODUCTION

Nonalcoholic fatty liver disease (NAFLD) is a metabolic disease, and it includes simple steatosis and nonalcoholic steatohepatitis; these diseases are related to obesity (Anstee et al., 2011). NAFLD is characterized by the accumulation of triglycerides and free fatty acids (FFA) in hepatocytes (Aray et al., 2004; Younossi et al., 2019). Obesity increases the release of FFA from adipose tissue to liver, contributing to the development of insulin resistance, and this decreases β‐oxidation by accumulating lipids in the liver (Zhang et al., 2018). FFAs can be incorporated into the synthesis of phosphatidylcholine, which can subsequently be derived into lysophosphatidylcholine (LPC) by the action of the enzyme phospholipase A_2_. An imbalance of LPC has been linked to the development of NAFLD in a model of rats with obesity and diabetes mellitus (Yang et al., 2018).

Despite the high prevalence of NAFLD, there are insufficient treatments, including few natural therapeutic alternatives. Green tea is one of the most consumed beverages in the world, and reports suggested that its consumption prevents the development of NAFLD (Zhou et al., 2019). Nam et al. (2018) reported that the administration of green tea extract to mice fed a high‐fat diet decreases the degree of NAFLD and increases the concentration of some liver LPCs such an LPC (16:1), while Sasaki et al. (2019) reported a decrease in liver levels of LPC (18:00), in male C57BL6/J mice subjected to a high‐fat diet. The extract of Roselle calyx is another beverage widely consumed with beneficial effects; Morales‐Luna et al. (2019) reported that the aqueous extracts of two varieties of *Hibiscus sabdariffa*, one with a high content of anthocyanins and the other without them but with a high content of organic acids, decreased the triglycerides accumulation of hepatocytes of rats fed with an HFF diet. Amaya‐Cruz et al. (2019) reported that the administration of Roselle calyx reduced insulin resistance and hepatic steatosis compared with rats fed an HFF diet. Other plant‐derived product employed is cinnamon (*Cinnamomum zeylanicum),* and it has been widely used as a food and beverage spreader. It was reported that an alcohol extract of cinnamon protects against alcohol‐induced fat accumulation in the liver of mice (Kanuri et al., 2009). Askari et al. (2014) reported that supplementation with cinnamon decreased some parameters related to NAFLD, such as the HOMA index, total triglycerides, and protein C reactive in patients with NAFLD. Another interesting plant is Malabar tamarind (*Garcinia gummi‐gutta*), and it can inhibit the enzyme citrate lyase, a key enzyme in the synthesis of fatty acids and cholesterol (Sripradha et al., 2016). It has been reported that the consumption of the extract of the fruit peel showed moderate‐fat infiltrations of the hepatocytes of rats fed with high cholesterol diet (Ates et al., 2012). *Mentha piperita* extracts ameliorated the steatosis score in diabetic rat liver, as well as glucose, total cholesterol, and triacylglycerides, parameters related to the development of NAFLD (Fig ueroa‐Pérez et al., 2015).

All these plants had improved the hepatic steatosis; however, the mechanisms of most of them have been little studied. Lipids, such as LPC, are essential molecules for biological processes as hepatic steatosis. Therefore, this study has the purpose to elaborate three functional beverages with extracts of Roselle (*Hibiscus sabdariffa*), peppermint (*Mentha piperita*), Malabar tamarind (*Garcinia gummi‐gutta*), cinnamon (*Cinnamomum zeylanicum*), and green tea (*Camellia sinensis*) and determine their effects on insulin resistance and hepatic steatosis, as well as phospholipids regulation in an animal model fed a high‐fat and fructose (HFF) diet.

## 2 MATERIALS AND METHODS

### 2.1 Materials

Dehydrated Roselle (*Hibiscus sabdariffa*) calyxes and cinnamon (*Cinnamomum zeylanicum*) were purchased from local markets (Queretaro, Mexico), whereas peppermint (*Mentha piperita*) plants were purchased from a local plant nursery (Floraplant S.A. de C.V., Celaya, Guanajuato, Mexico). Green tea extract was purchased from Döhler S.A de C.V (Mexico City, Mexico). Malabar tamarind extract (*Garcinia gummi‐gutta*) at 60% of hydroxycitric acid concentration was purchased from Future Foods S.A de C.V (Tlalnepantla, Mexico). Citric acid, sodium citrate, and potassium sorbate were purchased from Chemical Corporation JK S.A de C.V. (Guadalajara, Jalisco, Mexico).

The functional beverages used for the present study were identified as follows: beverage A (Red Roselle 2%, Green tea 1,125 g/L, Cinnamon 0.75 g/L, Malabar tamarind 0.8 g/L), beverage B (White Roselle 2%, Green tea 1,125 g/L, Cinnamon 1 g/L, Malabar tamarind 1.125 g/L), and beverage C (Green tea 1,5 g/L, Peppermint 0.5%, Malabar tamarind 1.5 g/L). All the beverages contained sucralose, acesulfame, citric acid, and potassium sorbate. These formulations were previously determined by sensory analysis.

### 2.2 Total polyphenol, flavonoid and anthocyanin content, antioxidant capacity

Total polyphenol content was determined according to Folin–Ciocalteu colorimetric method (Singleton & Rossi, 1965), and results were expressed as milligrams of gallic acid equivalents per dL (mg GAE dL^‐1^). Total flavonoid content was determined according to Liu and Hu (2002), and results were expressed as mg of (+)‐catechin equivalents per dL (mg CE dL^‐1^). Total anthocyanins were estimated through the pH differential method (Lee et al., 2005), and results were expressed as micrograms of delphinidin‐3‐glucoside equivalents per dL (mg D3GE dL^‐1^).

Antioxidant capacity was determined by two radical‐scavenging assays: 2,2’‐azino‐bis‐3‐ethylbenzothiazoline‐6‐sulfonic acid (ABTS) and 2,2‐diphenyl‐1‐picrylhydrazyl (DPPH) (Brand‐Williams et al., 1995; Re et al., 1999). Results were expressed as IC_50_ (half maximal inhibitory concentration) in mg/mL.

### 2.3 Treatments of animals

Male Wistar rats of 160–180 g were acquired from the National Autonomous University of Mexico (Juriquilla, Queretaro, Mexico), and maintained at 24 ± 1°C under a 12/12 hr light/dark cycle. Experiments on animals were performed in accordance with the Animal Care and Use Protocol of the Autonomous University of Queretaro, as recommended by the guidelines for animal testing (NOM‐062‐ZOO‐1999).

Groups of eight animals each were formed; the *SD* control group was fed a standard commercial diet (Rodent Lab chow 5,001, Purina Nutriments). The HFF control group and the three HFF‐treated groups (beverages A, B and C) were fed with diet standard supplemented with 18% lard and 16% fructose. Freshly prepared beverages were administered ad libitum overnight to the HFF‐treated groups, whereas tap water was administered to the control groups. After 19 weeks, the animals were anaesthetized, and blood was recovered by cardiac puncture; afterward, animals were sacrificed by exsanguination, livers, and adipose tissue were removed for further analysis. The blood was centrifuged at 3,000 x g for 5 min to obtain the serum and stored at −80°C until analysis. For biochemical analysis, livers were snap‐frozen and stored at −80ºC, whereas for histologic analysis, a fraction of livers and adipose tissue were stored in 10% neutral buffered formalin (pH 7.4). The experimental protocol was approved by the Bioethics Committee, Faculty of Chemistry, Autonomous University of Queretaro, CBQ16/0921‐2 (Querétaro, México).

### 2.4 Serum biochemical analysis

Serum glucose, triglycerides, total cholesterol, HD, LDL, AST, and ALT levels were measured using colorimetric commercial kits (Spinreact). Insulin was measured by ELISA kits (Millipore and Biosource).

### 2.5 Liver tissue histological analysis

Liver tissues were embedded in paraffin, sectioned at 5 μm, and stained with hematoxylin and eosin (H&E) solution. Samples were observed and photographed at 100 and 200X, analyzing ten images per animal. Hepatic steatosis degree was classified according to Burt scale: grade 0:0%–5%, grade 1:5%–33%, grade 2:33%–66%, and grade 3:66%–100% of hepatocytes with lipid vacuoles (Burt et al., 1998). The diameters of the adipocytes were measured with the Zen software of Carl Zeiss.

### 2.6 Hepatic triglycerides content

Lipids were extracted from frozen livers (100 mg) and determinate according to the method described by Fig ueroa‐Pérez et al. (2015).

### 2.7 LPC and LPE analysis

Frozen livers (50 mg) were homogenized with 50 μL of 0.15 M sodium chloride and 200 μL of chloroform:methanol (2:1) at 70,000 xg for 1 min. Afterward, samples were incubated for 60 min at −20°C and then were centrifuged at 11,200 xg for 5 min at 4°C, and the lower phases were recovered.

For the assessment of the lysophospholipids profile, an ultra‐performance liquid chromatography (UPLC) coupled with an electrospray ionization‐tandem mass spectrometer (ESI‐MS/MS) using a Xevo‐TQS system^®^ (Waters Co, USA) was used. An aliquot (1 μL) of hepatic lipid extract was injected in an Acquity BEH C18^®^ (10x50 mm, 1.7 mm) at 50°C. The solvent system (flow rate of 200 ml/min) used consisted of A (water containing 0.1% ammonium acetate and 0.1% formic acid) and B (acetonitrile: isopropanol 5:2 containing 0.1% ammonium acetate and 0.1% formic acid). Starting with 35% B, which was increased to 100% B in 6 min, and held for 7 min.

Mass spectrometry conditions were as follows: The source temperature was set at 120°C, and nitrogen was used as desolvation gas (800 L/h) at 250°C. The voltages of the sampling cone and capillary were 39 V and 3.2 kV, respectively. The chemical identification was performed by the analysis of fragmentation transitions for the Multiple Reaction Monitoring (MRM) using MassLynx software, which were obtained in positive mode. A standard of LPC (16:0) were used as fragmentation standard.

### 2.8 Statistical analysis

Data were expressed as mean values ± standard deviation. Statistical significance was determined by one‐way ANOVA. Differences between beverages were determined by comparison of means using Tukey's test (*p* < .05) for analysis. Differences between *SD* control and HFF treated with functional beverages, as well as HFF control and HFF treated with functional beverages, were determined by mean comparison using Dunnet test (*p* < .05) with JMP software (v11.0, SAS Institute).

Lipidomics data were normalized using an internal standard. Multivariate statistical analysis including PCA and OPLS‐DA, as well as heatmap, was carried out with Metaboanalyst 4.0 software (http://www.metaboanalyst.ca). The VIP (Variable importance in projection) values were determined to identify metabolites that contributed significantly to group separation in the PLSDA model. After that, a Dunnet test (*p* < .05) was used to compare differences between *SD* control and HFF treated with functional beverages, as well as HFF control and HFF treated with functional beverages.

## 3 RESULTS AND DISCUSSION

### 3.1 Phenolic compounds content of herb‐based beverages

Phenolic compounds are the main phytochemicals accessible in the diet, and they have preventive and therapeutic properties on insulin resistance and hepatic steatosis (Belwal et al., 2017; Ferramosca et al., 2017). The healthy benefits of the plant‐prepared functional beverages are related to their phytochemical composition, and mostly to its polyphenolic content and antioxidant capacity. The total polyphenols compounds were measured, beverage B gave the foremost value, showing a concentration up to 26.3% higher than beverage A (*p* < .05). Regarding to the flavonoids content, beverage C presented the highest values best up to 32.15% compared to beverage A (*p* < .05). Meanwhile, the anthocyanins were quantified only in beverage A (Table 1). The beverages reported in this paper are a good source of these phytochemicals, and all contain a high quantity of total polyphenols and flavonoids because the plants (Roselle, green tea, peppermint, and cinnamon) used to prepare them are rich in these compounds. The elevated concentration of total polyphenolic compounds by the B beverage is due to white Roselle, and it has a greater concentration of these compounds compare to red Roselle (Morales‐Luna et al., 2019). In addition, it contains green tea extract, which increased the concentration of them. Meanwhile, beverage C contains green tea extract and peppermint, its plants present more total polyphenols compounds content than Roselle, 37.6 and 26.1 ^1^mg GAE mL^‐1^ and flavonoids 51.3 and 22.6 mg CE mL^‐1^. Beverage A was the only one with anthocyanin, due to the addition of red Roselle.

**TABLE 1:** Total polyphenolic content and antioxidant activity of functional beverages

Assay	Beverage		
	A	B	C
Total phenols^1^	84.38 ± 0.37^c^	114.51 ± 2.16^a^	96.67 ± 1.92^b^
Total flavonoids^2^	8.61 ± 0.8^c^	10.43 ± 0.7^b^	12.69 ± 1.4^a^
Total anthocyanin^3^	24.21 ± 1.3	LDL	LDL
DPPH•^4^	13.5 ± 1.1^a^	8.9 ± 0.8^b^	9.2 ± 0.5^b^
ABTS•^+^^4^	11.4 ± 0.3^a^	9.6 ± 0.6^b^	10.4 ± 0.6^ab^

Note

Values were expressed as ^1^mg GAE dL^‐1^, ^2^mg CE dL^‐1^, ^3^mg D3GE dL^‐1^ y ^4^maximum effective concentration (IC_50;_ mg/mL). Data are expressed as mean ± standard deviation (*n* = 3). Different letters indicate significant statistical difference (*p* < .05), with the Tukey‐Kramer test.

Abbreviations: CE, catechin equivalents; D3GE, delphinidin‐3‐glucoside equivalents; GAE, gallic acid equivalents.

As mentioned, one possible mechanism to describe the beneficial effects of the plant‐based functional beverages is through their antioxidant capacity. It was determined by two methods (Table 1). The results are reported as the mean inhibitory concentration (IC_50_), which establishes the concentration of a substance necessary to inhibit the formation of the ABTS^+^ or DPPH^•^ radicals by 50%. Thus, lower IC_50_ values reflect a greater antioxidant capacity of beverages. According to the results, beverage B presented the highest antioxidant capacity for both assays, and these results are related to its major content of total polyphenols. Oxidative stress, the imbalance between free radicals and antioxidants, is a condition known associated with pathological processes such as diabetes, and hepatic steatosis (Fernández‐Sánchez et al., 2011). One of the major benefits of the consumption of natural products is the increase in the intake of phytochemicals with antioxidant properties. This fact contributes to limit the damage induced by the oxidative stress, produced by the hypercaloric diets through decoupling the cell‐oxidative metabolism, increasing reactive oxygen species (ROS), causing mitochondrial dysfunction which has been proposed as one of the first characteristics present in patients with NAFLD (Masarone et al., 2018).

### 3.2 Hepatic steatosis

**FIGURE 1:**
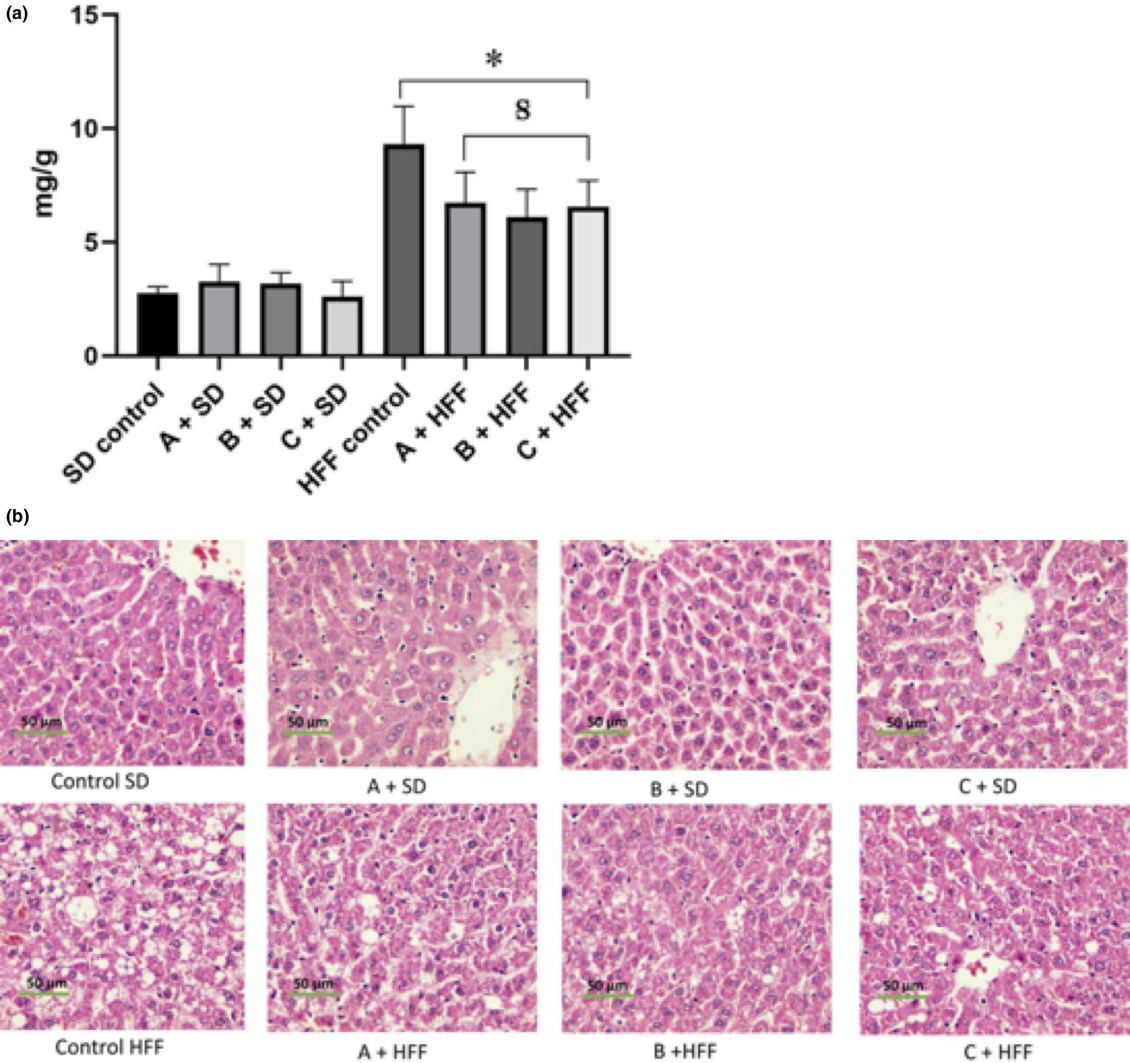
Effect of functional beverages on liver lipids. (a) quantification of liver triglycerides Data are expressed as mean ± standard deviation (*n* = 8). *Indicate statistical difference (*p* < .05) as compared to the *SD* control by Dunnet test. **^Ꞩ^** Indicate statistical difference (*p* < .05) between HFF control and groups HFF treated with beverages by Dunnet test. (b) Liver tissue microphotography stained with hematoxylin and eosin. *SD*, group fed with standard diet and HFF, group fed with high fat and fructose

The main objective of this work was the formulation of functional beverages for the treatment of hepatic steatosis, and the three beverages were tested in an animal model of HFF diet. The presence of steatosis was determined by an increase in the accumulation of fat in the liver. Such elevation was detected through a direct estimation of the triglycerides in the liver and liver histopathologic analysis. The HFF control group presented 3.3 times more triglycerides concentration in the liver than the *SD* control group (Figure 1a). The group fed with HFF diet and supplemented with any of the functional beverages showed a decrease in their triglycerides concentration, being group B + HFF the one that presented a greater decrease with 30.1%, followed by group C + HFF and A + HFF with a decrease of 25.2% and % 23.3% respectability (Figure 1a).

The changes in liver triglycerides content can be appreciated in the histological analysis showed in Figure 1b. The liver tissue from the *SD* control group does not present accumulation of lipids, nor the groups fed the same diet but supplemented with any of the functional beverages, and they were classified as grade 0 according to the Burt classification. The obese group shows vacuoles within the hepatocytes, being classified as grade 3, with vacuoles of fat in more than 66% of hepatocytes (Figure 1b). The group that consumed the beverage C was classified as grade 1 (<33% of hepatocytes with vacuoles). The groups that consumed the beverage A and B, the steatosis, were classified as grade 2 (33%–66% of hepatocytes with vacuoles) (Figure 1b). The triglyceride concentration in the groups HFF diet and beverages decreased, and it can be due to a lower absorption of triglycerides from the diet, a decrease in de novo synthesis or an increase in β‐oxidation. It has been reported that various compounds such as anthocyanins, catechins, epigallocatechin gallate, and curcumin can activate AMPK, and the active enzyme inhibits lipogenesis and increases β‐oxidation, which causes an inhibition in the accumulation of triglycerides in the liver (Kim et al., 2016). Another mechanism associated with the consumption of green tea in the improvement of NAFLD is to decrease the absorption of lipids from the diet (Xia et al., 2019).

The aspartate aminotransferase (AST) is an intracellular enzyme abundant in organs such as the heart and liver that catalyzes the reversible transfer of an amino group. Its high serum concentration is not specific for liver damage but suggests possible damage. Therefore, other enzymes such as the alanine aminotransferase (ALT) are measured, and this enzyme also transfers amino groups. Increased serum ALT is more specific and indicative of possible liver damage (Bayard et al., 2006).

In this study, we measured the activity of both AST and ALT enzymes. The activity of the AST increased slightly (1.2 times) in the animals consuming the HFF diet (Table 2). The animals fed with the HFF diet and consuming the beverages showed activities like the *SD* control animals (Table 2). In relation to ALT, the group fed with the HFF diet exhibited an activity 2.7 greater than the *SD* control group. The animals under HFF diet that consumed the beverages A or B improved the levels of the enzyme in serum, although they did not return to the basal levels; beverage C did not change the amount of circulating enzyme (Table 2). An unexpected result was the increase in the activity of ALT in serum in the animals fed with *SD* diet and consuming any of the beverages (Table 2).

**TABLE 2:** Effect of the consumption of functional beverages on serum lipid profile, triglycerides, and liver function enzymes activity in HFF‐diet‐fed obese rats

Parameters						
Experimental group	AST^1^	ALT^1^	Triglycerides^2^	HDL^2^	LDL^2^	Total cholesterol^2^
*SD*						
Control	28.7 ± 3.2	15.1 ± 6.3	103.52 ± 8.3	28.77 ± 5.1	13.17 ± 2.2	149.9 ± 6.3
A + *SD*	26.0 ± 4.4	19.4 ± 1.9*	98.38 ± 9.5	30.19 ± 5.9	15.23 ± 1.5	156.5 ± 11.3
B + *SD*	25.4 ± 2.9	21.3 ± 4.6*	96.80 ± 9.8	35.63 ± 7.4	14.95 ± 2.0	141.8 ± 16.3
C + *SD*	31.2 ± 5.4	25.6 ± 5.8*	99.49 ± 12.4	33.68 ± 7.9	14.42 ± 2.7	144.3 ± 11.7
HFF						
Control	34.6 ± 2.5	40.9 ± 6.0*	133.08 ± 20.2*	20.07 ± 6.0	18.74 ± 2.7	141.7 ± 10.5
A + HFF	24.4 ± 3.6^Ꞩ^	33.6 ± 7.8*	127.47 ± 22.2*	19.44 ± 5.7*	16.11 ± 1.5	151.0 ± 10.9
B + HFF	29.5 ± 4.3	30.9 ± 4.2*^,Ꞩ^	120.98 ± 13.6	23.65 ± 6.7	15.33 ± 1.8	147.9 ± 14.6
C + HFF	27.5 ± 5.9	38.5 ± 6.5*	115.15 ± 15.9	33.92 ± 7.5^Ꞩ^	16.97 ± 0.9	153.3 ± 13.5

Note

Values are expressed as ^1^UL^‐1^ and ^2^mg dl^‐1^. Data are expressed as mean ± standard deviation (*n* = 8). *Indicate statistical difference (*p* < .05) as compared to the *SD* control between beverages treated with *SD*, by Dunnet test. ^Ꞩ^ Indicate statistical difference (*p* < .05) between HFF control and groups HFF treated with beverages by Dunnet test.

Abbreviations: HFF, high fat and fructose; *SD*, standard diet.

Sanyal et al. (2015) reported a significant increase in AST and ALT in patients with NAFLD and insulin resistance compared to healthy patients, showing an increase of ALT, similar to our results. All functional beverages contained green tea extract, being beverage C the one with the major proportion, and this group showed the foremost value of ALT activity. Despite the reported benefits for green tea, it has been shown that high concentrations (approximately 1,300 mg total tea catechins, 800 mg EGCG) could induce liver damage, increasing ALT activity due to the pro‐oxidant property of tea catechins (Yu et al., 2017). Link up, it has reported that extract of *Hibiscus sabdariffa* reduced the activities of AST and ALT in the liver of diabetic rats, and this effect was partly related to its antioxidant activity associated with its flavonoids content (Adeyemi et al., 2014).

### 3.3 Lipid profile and insulin resistance

The consumption of diets high in fat and fructose induced a state of dyslipidemia characterized by high triglycerides, and LDL and low HDL levels in serum (Steinberger et al., 2009). Due to the lipolytic activity of visceral adipose tissue, free fatty acids increase in the blood stream, which are transported to the liver, thus increasing triglyceride synthesis.

As expected, the administration of an HFF diet to the rats induced an increase in the number of triglycerides in serum (Table 2), and the HFF‐fed animals had 28% more triglyceride than the *SD* control. Regard the groups consuming the beverages, they did not exhibit differences with any of the diets (Table 2). The exception was the group under HFF diet and consuming beverage C, and they presented a reduction of around 13%. Consumption of Roselle extract in different animal models and clinical studies was reported to influence the serum triglyceride levels, mainly attributed to compounds such as anthocyanins and protocatechuic acid (Da‐Costa‐Rocha et al., 2014). However, the effect seems to be related to the concentration of flavonoids, where beverage C has the major concentration of these phytochemicals, and it presented a decrease in the triglyceride levels; these compounds can decrease lipogenesis and increase ß‐oxidation. Additionally, it has been reported that they can inhibit the enzyme pancreatic lipase in a competitive way, thus reducing lipid absorption (Rahim et al., 2015).

The groups fed standard diet and supplemented with any of the functional beverages increased the serum quantities of HDL (Table 2). These numbers are commonly modified by HFF diets, and the control animals fed with this diet diminished the amount by around 30%. The groups consuming the beverages and fed with the HFF diet exhibited HDL numbers like the HFF control. Only the group under HFF diet and consuming beverage C shown an increase compared to the *SD* diet control group (Table 2). The increase in this parameter may be related to the higher concentration of flavonoids in beverage C, resulting from a higher concentration of green tea (33%) and to the addition of peppermint extract to beverage C, plants rich in flavonoids, compounds related to increased expression and activity of the enzyme paraoxonase 1 (PON1), which has been reported to increase HDL levels (Litvinov et al., 2012; Lou‐Bonafonte et al., 2015). For total and LDL cholesterol, no differences were observed in any of the groups.

Changes in the concentration of serum‐circulating glucose are one of the early indicators of an altered metabolism and health stage in animals fed with an HFF diet. In the present study, the obese control group presented a 22.4% higher glucose concentration than the healthy control group (Table 3). The consumption of the functional beverages, independently of the diet, did not present differences to their control group (Table 2). The regulation of circulating glucose in serum is tightly regulated, an increase in glucose stimulates the secretion of insulin by the pancreas. Analogous to the increase in glucose, the quantity of circulating insulin increased up to 318% in group‐fed HFF and the three functional beverages improved this parameter (27.7%–48.6%), being the beverage C who gives the best effect (Table 3).

**TABLE 3:** Effect of the consumption of functional beverages on glucose, insulin, HOMA index in rats fed with HFF

Beverage	Parameter		
	Glucose^1^	Insulin^2^	HOMA‐IR
Healthy control	112.64 ± 10.3	1.80 ± 0.5	12.1 ± 3.3
A + *SD*	125.31 ± 21.9	1.93 ± 0.7	17.3 ± 2.9
B + *SD*	110.03 ± 17.6	2.36 ± 0.3	14.2 ± 4.8
C + *SD*	115.54 ± 9.1	1.79 ± 0.6	12.5 ± 4.0
HFF control	139.70 ± 26.0	5.74 ± 1.0*	38.7 ± 5.9*
A + HFF	133.91 ± 21.5	4.15 ± 1.0*^,Ꞩ^	29.5 ± 4.5 *^,Ꞩ^
B + HFF	132.09 ± 22.1	3.23 ± 0.8*^,Ꞩ^	19.9 ± 4.4^Ꞩ^
C + HFF	129.44 ± 17.5	2.95 ± 0.7^Ꞩ^	24.4 ± 5.4*^,Ꞩ^

Note

Values are expressed as ^1^mg dl^‐1^, and ^2^ng ml^‐1^. Data are expressed as mean ± standard deviation (*n* = 8). *Indicate statistical difference (*p* < .05) as compared to the *SD* control by Dunnet test. ^Ꞩ^ Indicate statistical difference (*p* < .05) between HFF control and groups HFF treated with beverages by Dunnet test.

Abbreviations: HFF, group fed with high fat and fructose; *SD*, group fed with standard diet.

A sustained stimulation of the secretion of insulin eventually conducts to an insulin resistance stage. The estimate of the HOMA index gives an approximate value of this condition. The groups consuming standard diet and consuming any of the three functional beverages gave similar HOMA values (Table 3). The consumption of HFF diet increased 3 times this value in the control group. The inclusion of the functional beverages prevents, partially, the increase up to 50% in the group consuming beverage B (Table 3). Insulin resistance is an important parameter that has been related to the development of steatosis, and 70%‐80% of patients with obesity and diabetes have this condition (Kitade et al., 2017). Oxidative stress has been recognized as a key point to understand the development of this resistance, during the consumption of hypercaloric diets, different reactive oxygen species (ROS) are generated that can alter the organism's homeostasis through different routes such as: alteration of insulin receptor signaling (Hurrle & Hsu, 2017). Therefore, the consumption of beverages could be decreasing the formation of ROS, with beverage B having the highest antioxidant capacity and the greatest in improving insulin sensitivity.

### 3.4 LPC and LPE analysis

**FIGURE 2:**
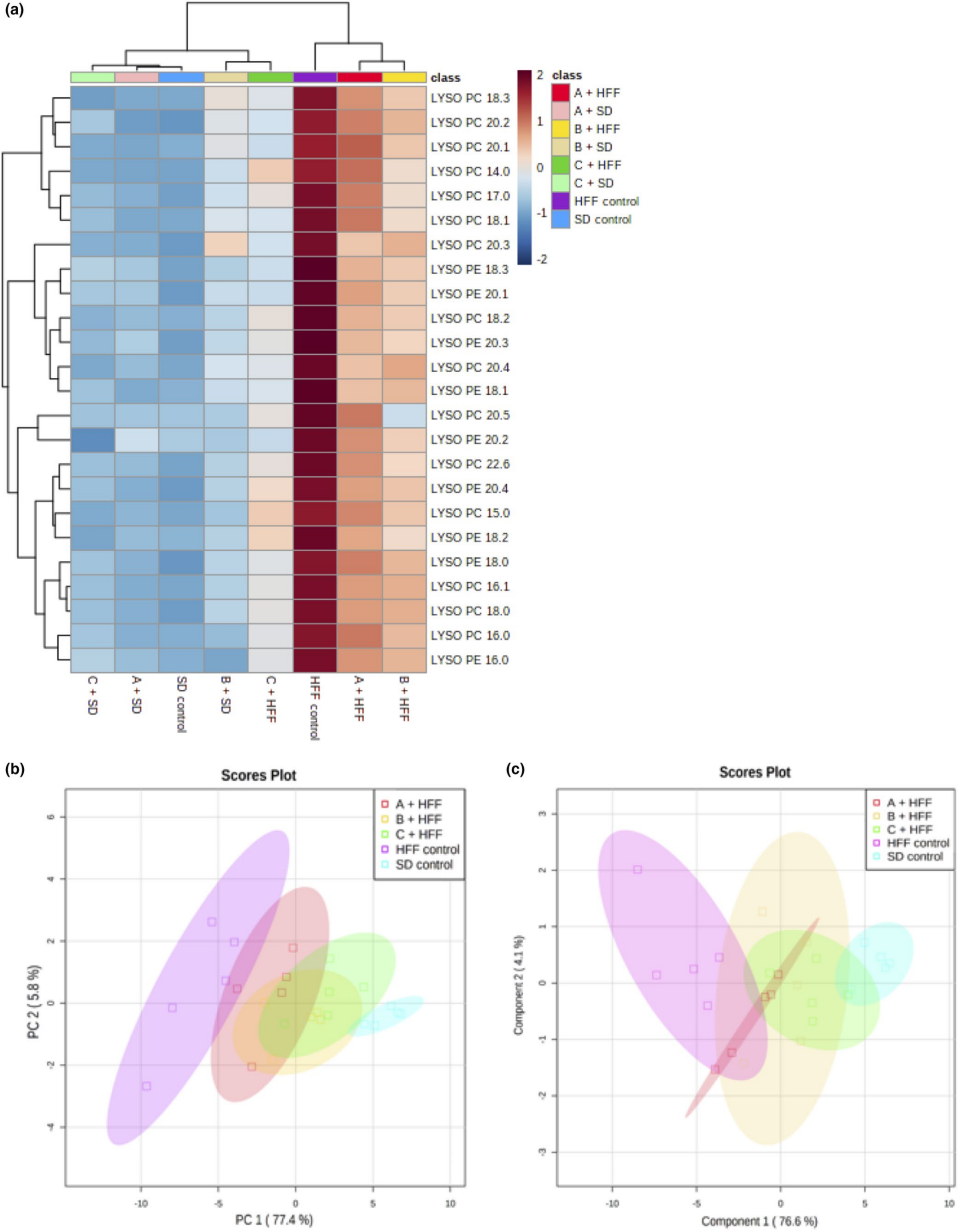
Multivariate analysis of liver lysophospholipids of rats fed with HFF and treated with functional beverages. Heat map (a), PCA plots analysis (b), and PLSDA plots analysis (c). Data analysis (*n* = 5). *SD*, group fed standard diet and HFF, group fed with high fat and fructose

High levels of some phospholipids have been associated with steatosis development; therefore, an analysis of some LPCs and LPEs was performed in this study. After UPLC MS/MS analysis, 15 different LPCs and 9 LPE were identified; subsequently, a multivariate analysis was performed on the MetaboAnalyst platform. The data were clustered and visualized in a heatmap, and it shows the differences between the experimental groups and their association according to an analysis of ANOVA and Euclidean distance. In Figure 2a, two main clusters were detected, one that included the groups fed with HFF, and other fed with *SD* diet and a group fed with HFF consuming beverage C. Interestingly, beverage consumption separates groups into a second cluster; animals fed with *SD*, A + *SD* and C + *SD*, another group that includes animals fed with B + *SD*, and C + HFF; finally, the group formed by B + *SD* and C + HFF.

Similar placement was observed for animals fed with *SD*, in agreement to previous results; therefore, the next analysis of these groups is not presented.

The multivariate analyses only were performed between the control groups and the groups with HFF diet and beverages. As a first step, we carry out a Principal Components Analysis (PCA) and multivariate technique and allow to visualize the variation and separation between the databases of experimental groups (Groth et al., 2013). The 82% of total of variance into the groups were represented by the first two principal components (PCs), where principal component 1 (PC1) and PC2 explained 6% and 76.8% of the variance, respectively (Figure 2b). The *SD* and HFF control groups are separated into different clusters, and the treated HFF groups are found between the clusters of the control groups, and the clusters of beverage B and C were closer to the *SD* control, and beverage A group closest to HFF control cluster. To discriminate the groups and explore the lysophospholipids more relevant in the study, a Partial Least Squares Discriminant Analysis (PLSDA) was performed. Comparable to PCA analysis, PLSDA shows a clusters separation between control groups, the cluster belonging to the group that consumed the beverage C was closer to the *SD* group, followed by beverage B (Figure 2c).

**FIGURE 3:**
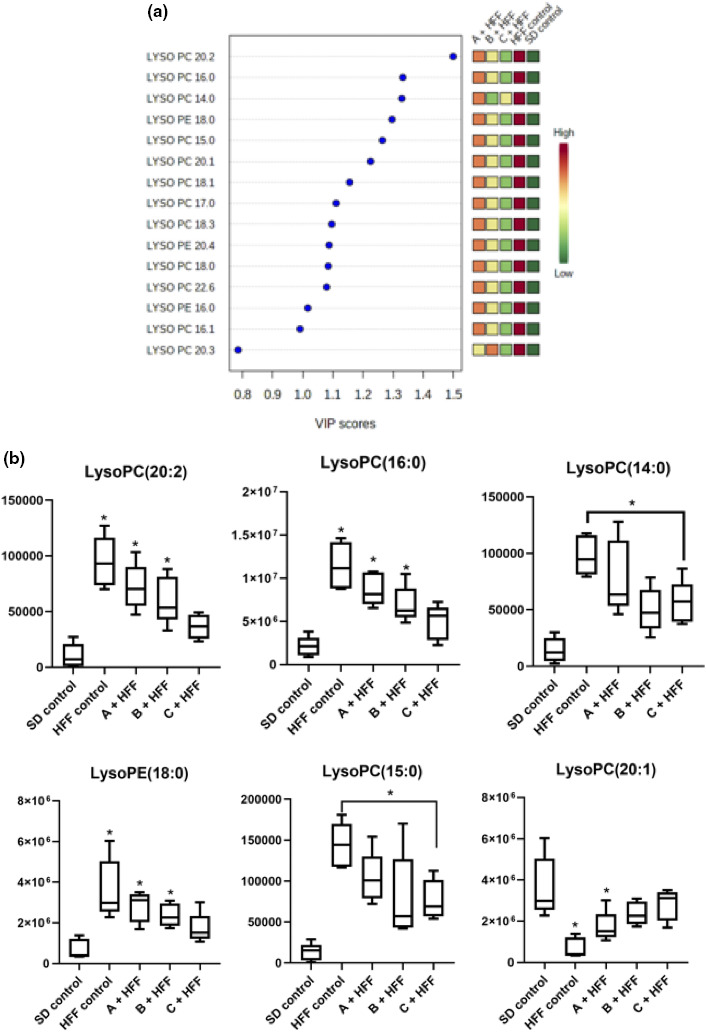
Lysophospholipids identified based on VIP scores in liver of rats fed with HFF and functional beverages. Partial least squares discriminant analysis [PLSDA] variable importance in projection [VIP] plot in liver (a), and Intensities of lysophospholipids (VIP > 1.2) in liver (b). Data analysis (*n* = 5). *SD*, group fed with standard diet and HFF, group fed with high fat and fructose. *Indicate statistical difference (*p* < .05) as compared to the *SD* control by Dunnet test

The statistical significance of the PLSDA classification model was assessed using a permutation test (100) with *p* < .01. Variable importance in projection (VIP) values are determined as a measure of the importance of each variable in the PLSDA model. The VIP scores show significant changes, and the LPCs saturated and unsaturated were higher in HFF control compared to *SD* control, an exception LPC 20:3. The group treated with beverages C had lower levels of these phospholipids, followed by beverages B (Figure 3a).

In addition, in this model 6 metabolites with VIP > 1.2 were identified: LysoPC (20:2), LysoPC (16:0), LysoPC (14:0), LysoPE (18:0), LysoPC (15:0), and LysoPC (20:1), and these compounds are mainly those that allow the separation in clusters of the experimental groups, and the change in the VIP score between the groups according to each molecule is shown in Figure 3b. It is observed that the animal fed with HFF diet increases the abundance of these compounds compared to the group fed with *SD* diet. By consuming the beverages, this effect is counteracted by decreasing the abundance, an effect marked mainly by beverage C, followed by beverage B. LPC and LPE are important signaling molecules involved in different processes such as inflammation and cell proliferation.

LPC is produced from phosphatidylcholine by hydrolysis with phospholipase A2 (PLA2), which removes one of the fatty acids. LPC is also formed by lecithin‐cholesterol acyltransferase activity caused by the increase of free fatty acids (FFA), produced by lipolysis. LPC‐induced lipoapoptosis in hepatocytes, ER stress, and inflammation producing NASH (Hirsova et al., 2016).

To our knowledge, there are no PLC regulation studies induced by phenolic compounds; however, for drugs, Yang et al. (2018) evaluated the administration of pioglitazone, agonist of PPAR in Male Otsuka Long‐Evans Tokushima Fatty and they observed that this drug improves the degree of hepatic steatosis, decreasing various LPCs, and this effect was associated with lower enzymatic activity of PLA_2_. Taken together, these data indicate that beverages significantly prevent HFF diet‐induced metabolite changes.

## 4 CONCLUSIONS

The consumption of natural beverages had a hepatoprotective effect, decreasing the degree of steatosis, being the beverages B and C, those with the significant effect, and both contain green tea and garcinia, being different for those beverages with red Roselle and the lowest content of garcinia compared to other beverages. The protection for steatosis was associated with an improve of insulin resistance and a decrease in the lysophosphatidylcholine content, principally for beverages C and B.

## CONFLICT OF INTEREST

Rubio‐Rodríguez Julio C, Reynoso‐Camacho Rosalia, Rocha Guzmán Nuria, and Salgado Luis M. declare that they do not have any conflict of interest. Experiments on animals were performed in accordance with the Animal Care and Use Protocol of the Autonomous University of Queretaro, as recommended by the guidelines for animal testing (NOM‐062‐ZOO‐1999).
